# Fuzzy Bayesian Network-Bow-Tie Analysis of Gas Leakage during Biomass Gasification

**DOI:** 10.1371/journal.pone.0160045

**Published:** 2016-07-27

**Authors:** Fang Yan, Kaili Xu, Xiwen Yao, Yang Li

**Affiliations:** School of Resources and Civil engineering, Northeastern University, Shenyang, Liaoning, P. R. China; Southwest University, CHINA

## Abstract

Biomass gasification technology has been rapidly developed recently. But fire and poisoning accidents caused by gas leakage restrict the development and promotion of biomass gasification. Therefore, probabilistic safety assessment (PSA) is necessary for biomass gasification system. Subsequently, Bayesian network-bow-tie (BN-bow-tie) analysis was proposed by mapping bow-tie analysis into Bayesian network (BN). Causes of gas leakage and the accidents triggered by gas leakage can be obtained by bow-tie analysis, and BN was used to confirm the critical nodes of accidents by introducing corresponding three importance measures. Meanwhile, certain occurrence probability of failure was needed in PSA. In view of the insufficient failure data of biomass gasification, the occurrence probability of failure which cannot be obtained from standard reliability data sources was confirmed by fuzzy methods based on expert judgment. An improved approach considered expert weighting to aggregate fuzzy numbers included triangular and trapezoidal numbers was proposed, and the occurrence probability of failure was obtained. Finally, safety measures were indicated based on the obtained critical nodes. The theoretical occurrence probabilities in one year of gas leakage and the accidents caused by it were reduced to 1/10.3 of the original values by these safety measures.

## Introduction

Biomass has been rapidly developed as a renewable energy source in recent years [1], and it has tremendous potential in solving future shortage of energy [2]. In China, the capacity of biomass energy increased from 2.2 GW [3] to 3 GW [4] between 2004 and 2015. Biomass energy usage is increasing in other countries as well [5–7]. As one of the most widely available energy sources [8], conversion of biomass resource includes biodiesel, biomass to liquid (BTL), biomass gasification, etc [9,10]. Recently, biomass gasification stations have been constructed and put into operation massively in rural areas of China. They are used to reduce the burning of crop straw, which causes air pollution [11]. And more importantly, agriculture wastes can be made into green energy *via* biomass gasification. However, hydrogen (H_2_), carbon monoxide (CO), and methane (CH_4_) which are produced by biomass gasification are flammable and CO has high poisonousness [12]; leakage of biomass gasification gas will lead to accidental fires and poisoning incidents [13,14]. Because the development and promotion of biomass gasification is restricted by their danger, therefore, PSA is necessary for biomass gasification system, and effective safety measures are needed to reduce the risks associated with gas leakage.

Biomass gasification system is complicated, various causes may lead to gas leakage, and gas leakage will cause multifarious consequences as well. Bow-tie analysis is a quantitative method which includes fault tree analysis (FTA) and event tree analysis (ETA, [15]). So the deduction and induction function of bow-tie analysis makes it possible to investigate the causes and consequences referred to gas leakage. As a concise and effective quantitative risk assessment (QRA) methodology, Paolo [16] applied bow-tie analysis to baseline risk assessment tool (BART), and they utilized bow-tie analysis for identification and assessment of any potential hazards and associated risks. Because bow-tie analysis can clearly display the links between causes, loss event (LE), conditional events (CE), and outcome events (OE), Chen [17] considered bow-tie analysis as an effective tool to identify environmental risk source. The application of bow-tie analysis is broad based on its characteristic. It can be applied in risk management of sea ports and offshore terminals [18], risk evaluation for natural gas industry [19], risk assessment of hexane distillation installation [20]. However, the focus of bow-tie analysis is displaying the whole scenario of accidents, identifying and assessing the potential causes and consequences. Causes which are more critical to the consequences based on the logical links and occurrence probabilities of themselves cannot be readily obtained by bow-tie analysis. Therefore, as a method widely used in PSA [21], bow-tie analysis can be mapped into BN to achieve the goal. Some research are involved in mapping bow-tie analysis into BN. Badreddine and Amor [22] took advantage of dynamic analysis function of BN to improve a bow-tie model. They constructed bow-tie diagrams in an automatic and dynamic way to implement the appropriate preventive and protective barriers in a dynamic system. Khakzad [23] made dynamic risk analysis of a physical reliability periodically updating system, the failure probabilities of safety barriers of bow-tie were periodically updated by using Bayesian theorem, and the probabilities of the consequences were estimated by the improved bow-tie analysis. They considered that the bow-tie's limitations resulting from its static constituents can be relaxed by mapping bow-tie analysis into BN [24]. Majeed [25] used bow-tie analysis and BN to confirm the critical elements of the well integrity model. In their study, the posterior probability was obtained by Bayesian theorem, then the critical elements were confirmed by the ratio of posterior probability to prior probability. All in all, bow-tie analysis mapping into BN can make dynamic risk analysis, and the critical elements of system can be confirmed by the calculation of posterior probability using Bayesian theorem. However, PSA importance measures can be introduced as well [26], because the conditional probability can be calculated by Bayesian theorem, BN can be fitted with these importance measures well, the importance measures can be computed accurately and easily. Thus the BN-bow-tie analysis is not only displaying the accidents scenarios of biomass gasification, but also making PSA to confirm the critical causes of accidents by adding the importance measures.

In order to make PSA of biomass gasification system, the reliability data is needed. Standard reliability data sources [27] can provide some common reliability data. Lopez [28] used the standard reliability data sources to confirm the probability of base event (BE) of FTA in liquefied natural gas (LNG) industry. Similarly, Khakzad [24] confirmed the failure probability in bow-tie analysis of a mixing tank system by referring to the standard reliability data sources. In addition, some reliability data cannot be obtained from currently available data. As fuzzy methods are widely used in risk analysis [29,30], fuzzy methods based on expert judgment can be the way to obtain reliability data [31,32]. However, fuzzy methods are practical and flexible in application for many fields. The fuzzy logic can be coupled with regression, nearest neighbor method, and artificial neural networks to construct a predictive model, and this model can be utilized effectively to make predicting demand for natural gas and energy cost savings in public buildings [33]. In Rodger's study [34], the fuzzy logic can cooperate with BN to implement probabilistic estimation, meanwhile, the method proposed by Rodger used the fuzzy clustering to produce a funnel diagram to make a clear and systematic demonstration for the relevance in supply chain backorder aging, unfilled backorders, and customer wait time. Moreover, Rodger [35] made a comprehensive study of group decision making, weighted average, linguistic terms, and fuzzy logic, in their study, a fuzzy induced linguistic ordered weighted averaging approach which can provide further insight and linguistic simplicity for decision makers was proposed to evaluate the risk in the supply chain. The fuzzy numbers reflect the linguistic expression of expert judgments to estimate events, for instance, if the expert judgment of a failure is 'about very low', the triangular number is introduced to indicate the judgment, and the trapezoidal number can indicate the judgment 'about very low to low'. Then fuzzy numbers can be converted to fuzzy failure rate (FFR, [36,37]), and the occurrence probability of failure is obtained. Ferdous [38] used triangular numbers to define expert judgments in the confirmation of occurrence probability in bow-tie analysis. In a fuzzy Bayesian network, triangular numbers were employed by Li [39] in quantitative human reliability analysis (HRA) frameworks. Ramzali [40] used expert judgment to obtain the failure probability of safety barriers in offshore drilling system, in their study, fuzzy numbers were introduced to reflect expert judgments. Various aggregation methods of fuzzy numbers are available. Bardossy [41] proposed a simple and effective approach to aggregate fuzzy numbers when they include only triangular or trapezoidal numbers. Hsu and Chen [42] proposed similarity aggregation method (SAM), when the fuzzy numbers were all triangular numbers or trapezoidal numbers, SAM was utility in aggregating fuzzy numbers with considering expert weighting [40,43]. Lin and Wang (Lin and Wang 1997) proposed an approach to aggregate fuzzy numbers including both triangular and trapezoidal numbers. However, fuzzy numbers based on expert judgment may be triangular or trapezoidal numbers, and the character of experts will affect their judgments as well. Therefore, in this study, Lin and Wang's method [44] was improved, the improved method can aggregate fuzzy numbers including both triangular and trapezoidal numbers, and expert weighting was also considered simultaneously. So it will make the occurrence probability of failure to be more objective, and the PSA of biomass gasification system to be more reliable.

This study identified the biomass gasification system by bow-tie analysis, causes of gas leakage and consequences resulted in gas leakage were obtained. Meanwhile, failure data was partly obtained from standard reliability data sources. For the failure data was not available from the existing data, fuzzy method based on expert judgment was employed to obtain the failure data. The fuzzy numbers which reflected the linguistic expression of expert judgments included both triangular and trapezoidal numbers, and an improved method was proposed to obtain the fuzzy failure data with considering the expert weighting. Then bow-tie analysis was mapping into BN (BN-bow-tie) to make PSA, three importance measures were introduced, and the critical nodes to accidents were obtained by computing the importance measures. Finally, safety measures aiming at the critical nodes were proposed, and the reduction of occurrence probabilities of accidents was calculated.

## Methods

### Model

**Fig 1:**
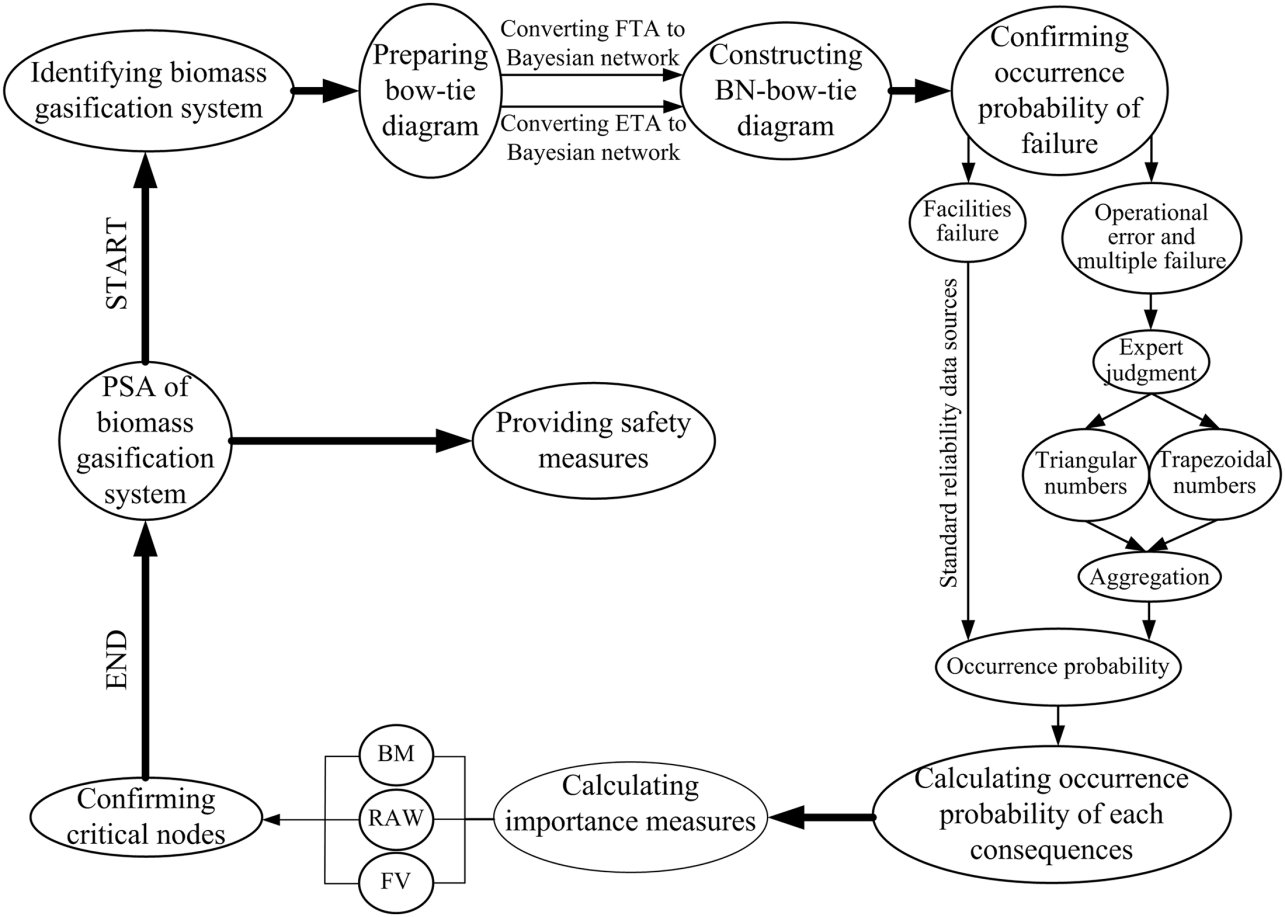
Proposed methodology.

Bow-tie analysis was used to display the accidents scenarios of a system from an LE. Causes of LE can be found by FTA of bow-tie analysis; consequences that result in LE can be identified by ETA of bow-tie analysis. In this proposed approach, FTA and ETA of bow-tie were transformed into BN to make the BN-bow-tie analysis. Units that give rise to accidents are more easily could be obtained. With this approach, the explicit occurrence probability of failure was needed. The occurrence probability of facilities failure was obtained from standard reliability data sources [27]. The occurrence probability of operational error cannot be confirmed from existing data. Then fuzzy methods based on expert judgment were used to achieve these occurrence probabilities. Subsequently, variable consequences were predicted by BN-bow-tie analysis. Finally, the critical nodes related to the consequences was obtained. Flowchart of the methodology was showed below (Fig 1).

### BN-bow-tie Analysis

Bow-tie was combined with FTA and ETA, and FTA and ETA were converted to BN. The algorithm of the logical relationship was identical to FTA and ETA. FTA in bow-tie was used to calculate LE as well as top event (TE) occurrence probability. If the occurrence probability of the basic event (BE) was obtained, the occurrence probability of the TE was also obtained. When the logical relationship of events was AND-gate, all events occured, and the TE occured. Eq 1 was used to calculate TE occurrence probability.

$$F_{TE}=\prod_{i=1}^{n}F_{BE_{i}}$$ (1)

If the logical relationship of events was OR-gate, only one of these events, the TE, was occurrence. Eq 2 was used to calculate TE occurrence probability.

$$F_{TE}\text{=1-}\prod_{i=1}^{n}\text{(1-}F_{BE_{i}})$$ (2)

The occurrence probability of TE of FTA was defined to be that of LE. Meanwhile, the occurrence probability of an initiating event (IE) of ETA was equal to that of LE. Eq 3 was used to calculate occurrence probability of OE in ETA.

$$F_{OE_{n}}=F_{IE}\cdot \prod_{i=1}^{n}P\text{(}E_{i}\text{=0)}\cdot \prod_{j=1}^{n}P\text{(}E_{j}\text{=1)}$$ (3)

BN is an inference approach that was combined with graph theory and probability theory. BN analysis of BN was based on the Bayesian theorem (Eq 4).

$$P(B|A)=\frac{P\text{(}A|B\text{)}\cdot P\text{(}B\text{)}}{P\text{(}A\text{)}}$$ (4)

According to the Bayesian theorem, three importance measures were introduced in the new methodology. So that events can be evaluated by their logical relationship and their occurrence probability in BN. The three importance measures were described below [26].

#### Birnbaum measure (BM)

BM measured increment of TE occurrence probability when BE occurred (Eq 5).

$$I_{BE}^{BM}=P(TE|BE=1)-P(TE|BE=0)$$ (5)

where *P*(*TE*|*BE* = 1) denoted the occurrence probability of TE when a BE occurred, *P*(*TE*|*BE* = 0) denoted the occurrence probability of TE when a BE didn't occur.

#### Risk achievement worth (RAW)

RAW evaluated the influence of BE for TE when it was considered with the occurrence probability of BE. RAW was adjusted by the occurrence probability of TE and BE. In this article, Eq 6 was used to calculate RAW.

$$I_{BE}^{RAW}=I_{BE}^{BM}\cdot \frac{P(BE)}{P(TE)}$$ (6)

where *P*(*BE*) denoted the occurrence probability of BE, *P*(*TE*) denoted the occurrence probability of TE under no conditions.

#### Fussel-Vesely (FV)

FV was used to describe the BE contribution to the failure of the system (Eq 7).

$$I_{BE}^{FV}=\frac{P(TE)-P(TE|BE=0)}{P(TE)}$$ (7)

In the proposed methodology, the critical nodes which triggered accidents more easily were obtained by calculating these importance measures.

### Confirming Occurrence Probability by Fuzzy Methods

In this approach, the occurrence probability of each BE was needed in BN-bow-tie analysis. BEs were divided into three classes: facilities failure, operational error and multiple failure, and multiple failure included facilities failure and operational error. The occurrence rate of facilities failure was obtained from standard reliability data resources, subsequently, occurrence rate was converted to occurrence probability by Eq 8. Because the occurrence probability of operational error and multiple failure could not be confirmed by existing data resources, the fuzzy methods based on expert judgment were used to estimate occurrence probabilities of operational error and multiple failure.

$$F=1-e^{-\lambda t}$$ (8)

where *F* denoted the occurrence probability of failure, *λ* denoted the occurrence rate of failure.

The fuzzy methods involved aggregating the different judgment of different experts, expert weighting was considered and triangular fuzzy numbers or trapezoidal fuzzy numbers proposed by experts were estimated to calculate FFR [44], and FFR was converted to the occurrence probability of operational error and multiple failure.

The following steps were used to confirm occurrence probability based on expert judgment.

1. Confirm the weighting of each expert. The weighting of each expert was partitioned by age, education background, years of service, and professional position (Table 1, [40,43]).

**Table 1:** Weighting Score of Different Expert Factors.

Factors	Classification	Weighting Score
Age (*a*)	< 30 years old	1
	30–39 years old	2
	40–49 years old	3
	≥ 50 years old	4
Education background(*b*)	High school	1
	Junior college	2
	Bachelor	3
	Master	4
	PhD	5
Service year (*c*)	< 5 years	1
	5–10 years	2
	11–20 years	3
	21–30 years	4
	≥ 30 years	5
Professional Position (*d*)	Worker	1
	Technician	2
	Junior academic/engineer	3
	Senior academic/engineer	4

Eq 9 showed the calculation of the total weighting score of each expert, and the weighting of each expert was calculated by Eq 10.

$$S_{u}=S_{u_{a}}+S_{u_{b}}+S_{u_{c}}+S_{u_{d}}$$ (9)

where *S*_*u*_ denoted the total weighting score of an expert.

$$W_{u}=\frac{S_{u}}{\sum_{u=1}^{M}S_{u}}$$ (10)

where *M* denoted the number of experts.

2. Judgment of occurrence probabilities were classified as nonoccurrence, absolute low, very low, low, fairly low, medium, fairly high, high, very high, absolute high or occurrence. The level value of each classification were defined from 0 to 1 (Table 2, [29,45]). Then corresponding triangular numbers or trapezoidal numbers proposed by experts were used to judge occurrence probabilities of events.

**Table 2:** Level Value of Each Classification.

Judgment of Occurrence Probabilities Classification	Level Value
Nonoccurrence	0
Absolute low	0.1
Very low	0.2
Low	0.3
Fairly low	0.4
Medium	0.5
Fairly high	0.6
High	0.7
Very high	0.8
Absolute high	0.9
Occurrence	1

3. Aggregate the fuzzy numbers. When the fuzzy numbers were all triangular number or trapezoidal number, Eq 11 was used to calculate the aggregated fuzzy number of experts for one event [42].

$${\tilde{A}}_{aggregated}=\sum_{u=1}^{M}W_{u}⊗{\tilde{A}}_{u}$$ (11)

where ${\tilde{A}}_{aggregated}$ denoted the aggregated fuzzy numbers of *M* experts' judgment, *W*_*u*_ denoted the weighting of expert.

Assume ${\tilde{A}}_{u}=(a_{u1},a_{u2},a_{u3})$ was triangular number and ${\tilde{A}}_{u}=(a_{u1},a_{u2},a_{u3},a_{u4})$ was trapezoidal number, calculation of $W_{u}⊗{\tilde{A}}_{u}$ was showed as below (Eqs 12 and 13). Then Eqs 14 and 15 were used to calculate the value of ${\tilde{A}}_{1}⊕{\tilde{A}}_{2}$ [30].

$$W_{u}⊗{\tilde{A}}_{u}=(W_{u}\times a_{u1},W_{u}\times a_{u2},W_{u}\times a_{u3})$$ (12)

$$W_{u}⊗{\tilde{A}}_{u}=(W_{u}\times a_{u1},W_{u}\times a_{u2},W_{u}\times a_{u3},W_{u}\times a_{u4})$$ (13)

$${\tilde{A}}_{1}⊕{\tilde{A}}_{2}=(a_{11}+a_{21},a_{12}+a_{22},a_{13}+a_{23})$$ (14)

$${\tilde{A}}_{1}⊕{\tilde{A}}_{2}=(a_{11}+a_{21},a_{12}+a_{22},a_{13}+a_{23},a_{14}+a_{24})$$ (15)

4. If the fuzzy numbers included both triangular number and trapezoidal number. Algorithm of the aggregation proposed by Lin and Wang [44] was employed, furthermore, experts weighting were considered to improve the method. The following example was introduced to illustrate the algorithm.

Assume ${\tilde{A}}_{1}=(a_{11},a_{12},a_{13})$ was triangular number proposed by expert 1 and ${\tilde{A}}_{2}=(a_{21},a_{22},a_{23},a_{24})$ was trapezoidal number proposed by expert 2. Experts weightings of them were *W*_*1*_ and *W*_*2*_, respectively. The membership function of them were;

$$f_{{\tilde{A}}_{1}}(x)=\{\begin{array}{ll} (x-a_{11})/(a_{12}-a_{11}), & a_{11}\leq x\leq a_{12}\\ (a_{13}-x)/(a_{13}-a_{12}), & a_{12}\leq x\leq a_{13}\\ 0, & \text{otherwise} \end{array}$$

$$f_{{\tilde{A}}_{2}}(y)=\{\begin{array}{ll} (y-\;a_{21})/(a_{22}-\;a_{21}), & a_{21}\leq y\leq a_{22}\\ 1,\; & a_{22}\leq y\leq a_{23}\\ (a_{24}-y)/(a_{24}-a_{23}), & a_{23}\leq y\leq a_{24}\\ 0, & \text{ otherwise} \end{array}$$

α-cut method [44,46] was employed to aggregate the fuzzy numbers, meanwhile, expert weighting was also considered (Eq 16).

$${\tilde{A}}_{W_{\alpha }}=\sum_{u=1}^{n}W_{u}⊗{\tilde{A}}_{u_{\alpha }}$$ (16)

where ${\tilde{A}}_{W_{\alpha }}$ denoted α-cut for membership function of the aggregated fuzzy number ${\tilde{A}}_{W}$, *W*_*u*_ denoted expert weighting, ${\tilde{A}}_{u_{\alpha }}$ denoted α-cut for membership function of ${\tilde{A}}_{u}$, *n* was the number of fuzzy numbers.

Then α-cut for membership function of ${\tilde{A}}_{1}$ and ${\tilde{A}}_{2}$ were;

$$\left\{ \begin{array}{l} {\tilde{A}}_{1_{\alpha }}=[x_{1},x_{2}]\\ {\tilde{A}}_{2_{\alpha }}=[y_{1},y_{2}] \end{array}\right.$$

Set *α* = (*x−a*_11_)/(*a*_12_*−a*_11_), and *x* can be either *x*_1_ or *x*_2_, so it can be obtained that *x*_1_ = (*a*_12_−*a*_11_)α+*a*_11_, and by this analogy, the α-cut values of ${\tilde{A}}_{1}$ and ${\tilde{A}}_{2}$ was calculated as;

$$\left\{ \begin{array}{l} x_{1}=(a_{12}-a_{11})\alpha +a_{11}\\ x_{2}=a_{13}-(a_{13}-a_{12})\alpha \\ y_{1}=(a_{22}-a_{21})\alpha +a_{21}\\ y_{2}=a_{24}-(a_{24}-a_{23})\alpha \end{array}\right.$$

${\tilde{A}}_{W_{\alpha }}$ was computed as (Eqs 12 through 16);

$$\begin{array}{ll} {\tilde{A}}_{W_{\alpha }} & =W_{1}⊗{\tilde{A}}_{1_{\alpha }}⊕W_{2}⊗{\tilde{A}}_{2_{\alpha }}\\ & =W_{1}⊗[x_{1},x_{2}]⊕W_{2}⊗[y_{1},y_{2}]\\ & =W_{1}⊗[(a_{12}-a_{11})\alpha +a_{11},a_{13}-(a_{13}-a_{12})\alpha ]\\ & \;⊕W_{2}⊗[(a_{22}-a_{21})\alpha +a_{21},a_{24}-(a_{24}-a_{23})\alpha ]\\ & =[(W_{1}(a_{12}-a_{11})+W_{2}(a_{22}-a_{21}))\alpha +W_{1}a_{11}+W_{2}a_{21},\\ & \;W_{1}a_{13}+W_{2}a_{24}-(W_{1}(a_{13}-a_{12})+W_{2}(a_{24}-a_{23}))\alpha ] \end{array}$$

Set ${\tilde{A}}_{W_{\alpha }}=[z_{1},z_{2}]$, we can obtained that;

$$\left\{ \begin{array}{l} \alpha =\frac{z_{1}-(W_{1}a_{11}+W_{2}a_{21})}{W_{1}(a_{12}-a_{11})+W_{2}(a_{22}-a_{21})}\\ \alpha =\frac{W_{1}a_{13}+W_{2}a_{24}-z_{2}}{W_{1}(a_{13}-a_{12})+W_{2}(a_{24}-a_{23})} \end{array}\right.$$

Then, the membership function of the aggregated fuzzy number can be obtained;

$$f_{{\tilde{A}}_{W}}(z)=\{\begin{array}{ll} \frac{z-(W_{1}a_{11}+W_{2}a_{21})}{W_{1}(a_{12}-a_{11})+W_{2}(a_{22}-a_{21})}, & W_{1}a_{11}+W_{2}a_{21}\leq z\leq W_{1}a_{12}+W_{2}a_{22}\\ 1, & W_{1}a_{12}+W_{2}a_{22}\leq z\leq W_{1}a_{12}+W_{2}a_{23}\\ \frac{W_{1}a_{13}+W_{2}a_{24}-z}{W_{1}(a_{13}-a_{12})+W_{2}(a_{24}-a_{23})}, & W_{1}a_{12}+W_{2}a_{23}\leq z\leq W_{1}a_{13}+W_{2}a_{24}\\ 0,\; & \text{otherwise} \end{array}$$

After that, the aggregated fuzzy number ${\tilde{A}}_{W}$ of ${\tilde{A}}_{1}$ and ${\tilde{A}}_{2}$ was achieved;

$${\tilde{A}}_{W}=(W_{1}a_{11}+W_{2}a_{21},W_{1}a_{12}+W_{2}a_{22},W_{1}a_{12}+W_{2}a_{23},W_{1}a_{13}+W_{2}a_{24})$$

Moreover, when the number of fuzzy numbers was more than two, aggregation algorithm was similar to the procedure above.

5. After the aggregated fuzzy number was confirmed, centroid-index method (Eq 17, [36]) was used to deal with the fuzzy number, then the fuzzy possibility score (FPS) was obtained. Assume ${\tilde{A}}_{u}=(a_{u1},a_{u2},a_{u3})$ was triangular number and ${\tilde{A}}_{u}=(a_{u1},a_{u2},a_{u3},a_{u4})$ was trapezoidal number. Eq 18 was used to calculate FPS when fuzzy number was triangular number, and Eq 19 was used to calculate trapezoidal number.

$$X=\frac{\int g(x)xdx}{\int g(x)dx}$$ (17)

where *X* is the defuzzified output, *g*(*x*) is the membership function, and *x* is the output variable.

$$FPS=\frac{1}{3}(a_{u1}+a_{u2}+a_{u3})$$ (18)

$$FPS=\frac{1}{3}\frac{{\left(a_{u3}+a_{u4}\right)}^{2}-a_{u3}a_{u4}-{\left(a_{u1}+a_{u2}\right)}^{2}+a_{u1}a_{u2}}{\left(a_{u4}+a_{u3}-a_{u1}-a_{u2}\right)}$$ (19)

6. Finally, Eq 20 converted the FPS to FFR [37], and FFR was converted to occurrence probability of operational error and multiple failure (Eq 8).

$$FFR=\{\begin{array}{r} \begin{array}{cc} \begin{array}{l} 1/10^{k},\;\\ 0, \end{array} & \begin{array}{r} FPS\neq 0\\ FPS=0 \end{array} \end{array}\\ k=2.301\times {\left[(1-FPS)/FPS\right]}^{1/3} \end{array}$$ (20)

## Results

### Biomass Gasification System

**Fig 2:**
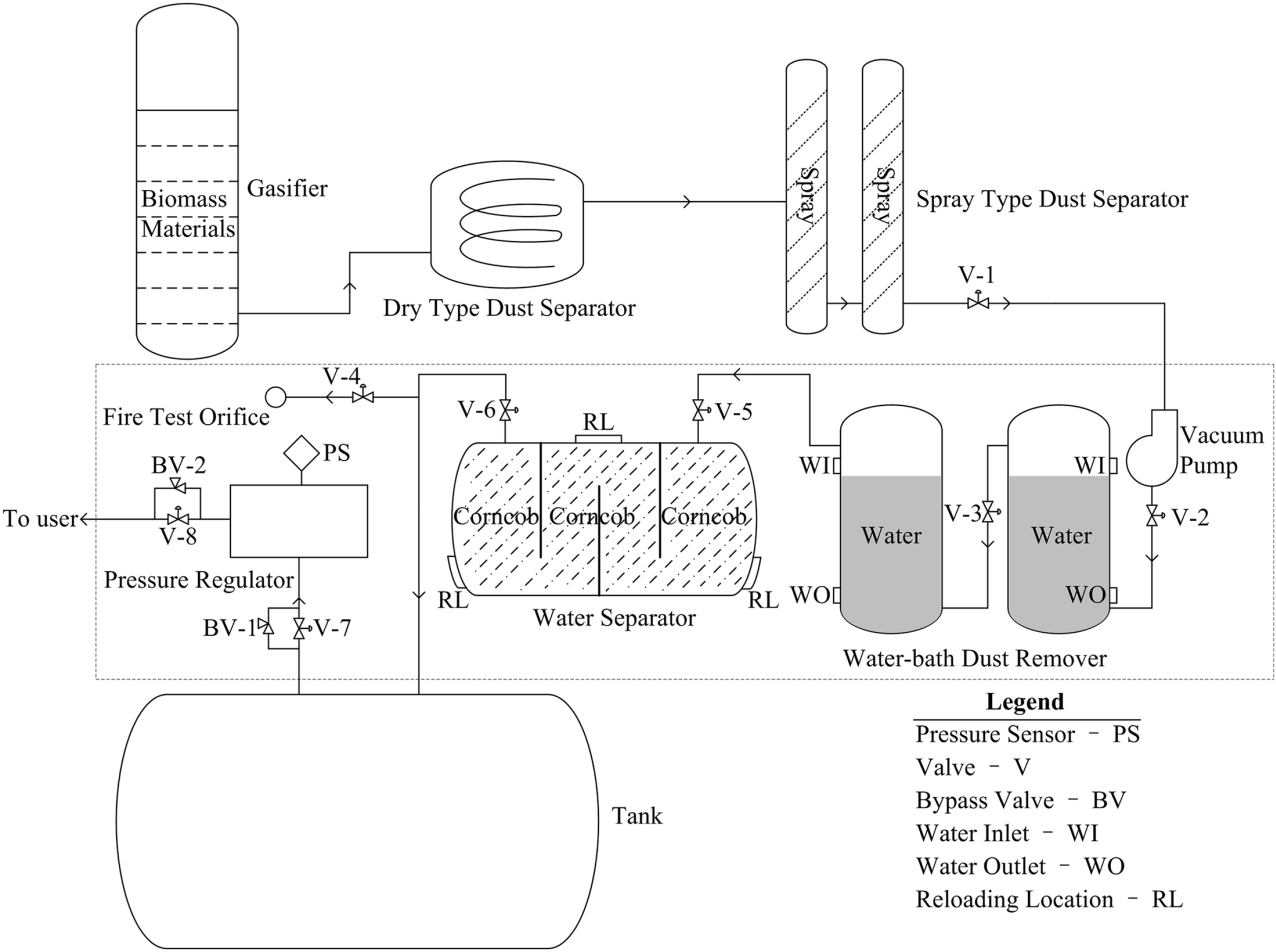
Biomass gasification system.

The biomass gasification system included a gasifier, dry type dust separator (DTDS), spray type dust separator (STDS), vacuum pump (VP), water-bath dust remover (WBDR), water separator (WS), tank, and pressure regulator (PR) (Fig 2). Biomass materials were burned in the gasifier with insufficient oxygen, and biomass gasses (hereafter referred to as “gas”) including CO, H_2_ and CH_4_ were produced by chemical reactions. The gas went into the DTDS, where most dust was separated. The VP was located between the STDS and WBDR. The gas was flowed into the STDS by the VP and was cleaned by the spray in the STDS. The WBDR provided further decontamination. Valve 2 (V-2) controlled the input of gas for the first WBDR, and valve 3 (V-3) controlled another. There was a water inlet (WI) and water outlet (WO) on the WBDR, and water in the WBDR was replaced through WI and WO. Waste water was discharged from the WO, and fresh water was injected into the WBDR from the WI such that the liquid level was below the WI. After the WBDR, the gas arrived at the WS, where the inlet and outlet were controlled by valve 5 (V-5) and valve 6 (V-6). Residual water in the gas was absorbed by corncobs in the WS, which were replaced *via* three reloading locations (RL). There was a fire test orifice (FTO) setting after WS that tested the ignitability of gas at the beginning of production; FTO was controlled by valve 4 (V-4). Finally, the cleaned gas was stored in an external tank. Gas was released from the tank into the PR, which contained a bypass valve 1 (BV-1) installed in parallel with valve 7 (V-7) of the PR. BV-1 ensured that if V-7 was plugged, gas in tank would be released to maintain a safe pressure level. The pressure was monitored by a pressure sensor (PS). Valve 8 (V-8) was located after the PR, and bypass valve 2 (BV-2) was installed in parallel with V-8. Hence, when V-8 was plugged, the gas was transferred from BV-2.

### Analysis of Gas Leakage in the Biomass Gasification System

**Fig 3:**
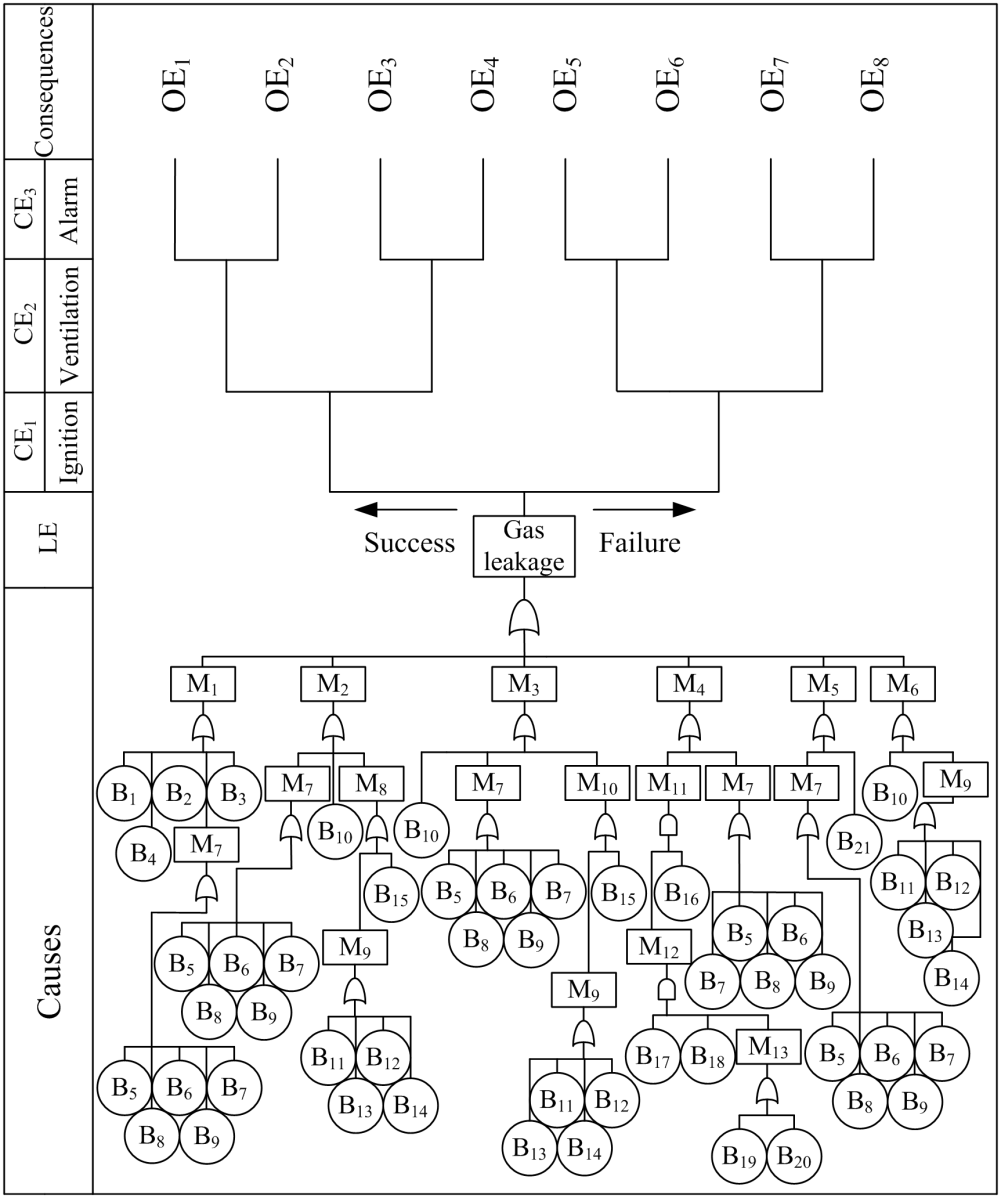
Diagram of bow-tie analysis for gas leakage.

As mentioned previously, the devices and pipelines before and after the VP were under the condition of negative and positive pressure during production process, respectively. No leakage could occur in the areas with the condition of negative pressure. And the tank was external to the system, then the parts where gas leakage would be considered were encircled by the dashed line in Fig 2. Gas leakage was set as the TE to make FTA. Meanwhile, ventilation system and alarm system were placed in the system. Then set them and ignition as CEs, and gas leakage was set as the IE to make ETA. Various OEs were obtained by the conditions of the CEs. Finally, gas leakage was set to be the LE, bow-tie analysis connected the FTA and ETA by the LE (Fig 3, Table 3). Eight OEs were predicted depending on the success or failure of CEs (Fig 3, Table 4). Gas ignition would occur if CE_1_ was success but not if CE_1_ was failure.

**Table 3:** Details of Bow-tie Components in Fig 3.

Event	Symbol	Logic Link Type	Failure Model
Gas leakage	Gas leakage	OR-gate	------
Gas leakage of VP	M_1_	OR-gate	------
Gas leakage of WBDR	M_2_	OR-gate	------
Gas leakage of WS	M_3_	OR-gate	------
Gas leakage of PR	M_4_	OR-gate	------
Gas leakage of FTO	M_5_	OR-gate	------
Gas leakage of pipelines	M_6_	OR-gate	------
Gas leakage of valve	M_7_	OR-gate	------
Gas leakage of WI	M_8_	OR-gate	------
Flange failure	M_9_	OR-gate	------
Gas leakage of RL	M_10_	OR-gate	------
Gas leakage by high pressure	M_11_	AND-gate	------
High pressure	M_12_	AND-gate	------
Manual pressure relief failure	M_13_	OR-gate	------
VP Seal Failure	B_1_	------	Facilities failure
Cylinder liner failure	B_2_	------	Facilities failure
Breakage of VP	B_3_	------	Facilities failure
Wear of VP	B_4_	------	Facilities failure
Valve Seal Failure	B_5_	------	Facilities failure
Gaskets failure	B_6_	------	Facilities failure
Seat rings failure	B_7_	------	Facilities failure
Breakage of valve	B_8_	------	Facilities failure
Wear of valve	B_9_	------	Facilities failure
Material failure	B_10_	------	Facilities failure
Breakage of flange	B_11_	------	Facilities failure
Erosion	B_12_	------	Facilities failure
Looseness	B_13_	------	Facilities failure
Wear of flange	B_14_	------	Facilities failure
Flange isn't tightly clipped	B_15_	------	Operational error
Leakage	B_16_	------	Facilities failure
V-8 plugged	B_17_	------	Facilities failure
BV-2 is failed to open on demand	B_18_	------	Facilities failure
Operational error	B_19_	------	Operational error
PS is failed to function on demand	B_20_	------	Facilities failure
V-4 is not closed	B_21_	------	Operational error
Ignition	CE_1_	------	Multiple failure
Ventilation	CE_2_	------	Multiple failure
Alarm	CE_3_	------	Multiple failure

**Table 4:** OE Analysis.

Event	Symbol	Event	Symbol
Gas stays in room briefly	OE_1_	Poisoning, minor casualties	OE_2_
Fire, minor property damage	OE_3_	Fire and poisoning, major property damage, major casualties	OE_4_
Gas stays in room briefly	OE_5_	Poisoning, minor casualties	OE_6_
Gas accumulation in room	OE_7_	Poisoning, major casualties	OE_8_

### BN-Bow-Tie Model of Gas Leakage

**Fig 4:**
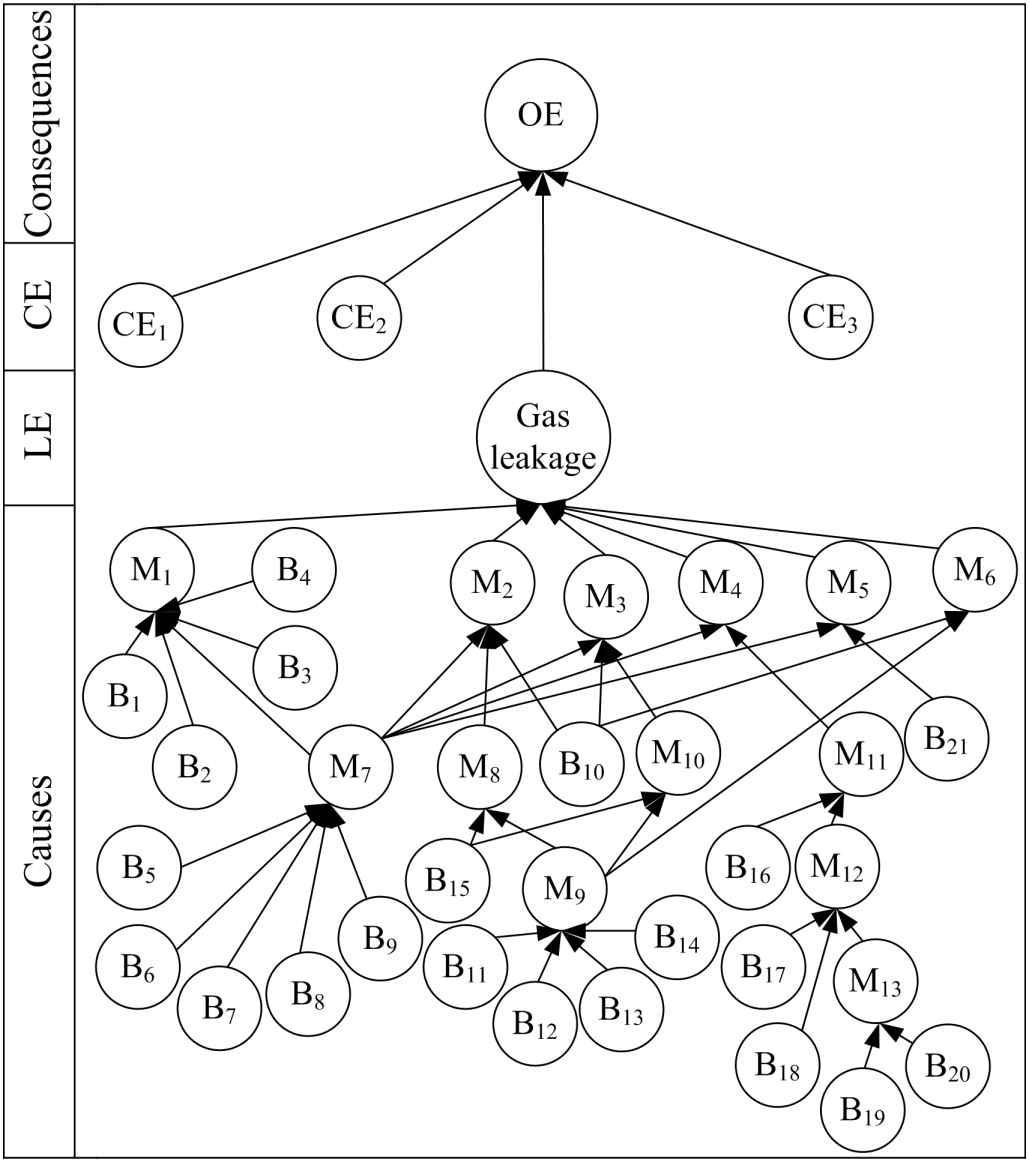
BN-bow-tie diagram of gas leakage.

The BN-bow-tie model of gas leakage was established by converting FTA and ETA into BN (Fig 4).

### Confirming Occurrence Probability of Each Basic Event and Conditional Event

Occurrence probability of facilities failure in the biomass gasification system was retrieved from a standard reliability data resource (Eq 8, Table 5, [27]).

**Table 5:** Occurrence Probability of Facilities Failure.

Event	Symbol	Occurrence Rate (d^-1^)	Occurrence Probability (in 1 year)
VP Seal Failure	B_1_	4.443e-4	1.497e-1
Cylinder Liner Failure	B_2_	1.018e-5	3.709e-3
Breakage of VP	B_3_	8.384e-5	3.014e-2
Wear of VP	B_4_	6.787e-4	2.194e-1
Valve Seal Failure	B_5_	4.305e-6	1.570e-3
Gasket Failure	B_6_	6.578e-6	2.398e-3
Seat Ring Failure	B_7_	5.038e-6	1.837e-3
Breakage of valve	B_8_	1.900e-6	6.933e-4
Wear of valve	B_9_	6.242e-6	2.276e-3
Material Failure	B_10_	2.942e-6	1.073e-3
Breakage of flange	B_11_	9.660e-7	3.525e-4
Erosion	B_12_	1.194e-5	4.349e-3
Looseness	B_13_	3.956e-5	1.434e-2
Wear of Flange	B_14_	1.142e-5	4.160e-3
Leakage	B_16_	1.332e-4	4.746e-2
V-8 Plugged	B_17_	1.462e-8	5.336e-6
BV-2 Fails to Open on Demand	B_18_	5.040e-6	1.838e-3
PS Fails to Function on Demand	B_20_	6.660e-5	2.402e-2

Note: all data was obtained from OREDA (2002) [27].

The other occurrence probabilities were confirmed by expert judgment. Five experts were invited to make judgment (Table 6). The weighting of each expert was calculated by Table 1 and Eqs 9 through 10 (Table 7).

**Table 6:** Description of Experts.

Expert	Age (years)	Education	Service (years)	Professional Position
1	55	High school	22	Worker
2	36	Master	11	Technician
3	40	Bachelor	18	Junior engineer
4	47	PhD	14	Senior engineer
5	50	PhD	21	Senior academic

**Table 7:** Weighting of Experts.

Expert	Weighting Score				Total Weighting Score	Weighting
	Age	Education	Service	Professional Position		
1	4	1	4	1	10	0.154
2	2	4	3	2	11	0.169
3	3	3	3	3	12	0.185
4	3	5	3	4	15	0.231
5	4	5	4	4	17	0.261
Total	16	18	17	14	65	1

Each expert gave judgment based on Table 2 to the events which belonged to the failure mode of operational error or multiple failure, and the corresponding fuzzy numbers were obtained (Table 8). Then the fuzzy numbers were aggregated by Eqs 11 through 16 (Table 9). Finally, the aggregated fuzzy numbers were converted to FPS and FFR by Eqs 17 through 20 (Table 9), and FFR was converted to occurrence probability by Eq 8 (Table 9).

**Table 8:** Experts Judgment of Operational Error and Multiple Failure.

Event	Fuzzy numbers proposed by each expert		
Flange is not tightly clipped (B_15_)	Expert1	Expert 2	Expert 3
	(0.2,0.3,0.4,0.5)	(0.1,0.2,0.3,0.5)	(0.2,0.3,0.4,0.5)
	Expert 4	Expert 5	
	(0.1,0.2,0.3,0.4)	(0.1,0.2,0.3,0.5)	
Operational error (B_19_)	Expert 1	Expert 2	Expert 3
	(0.1,0.2,0.3,0.5)	(0.2,0.3,0.4)	(0.1,0.3,0.4,0.5)
	Expert 4	Expert 5	
	(0.2,0.3,0.4,0.5)	(0.2,0.3,0.5)	
V-4 is not closed (B_21_)	Expert 1	Expert 2	Expert 3
	(0.2,0.3,0.4)	(0.1,0.3,0.4,0.5)	(0.1,0.2,0.3,0.4)
	Expert 4	Expert 5	
	(0.1,0.3,0.5)	(0.1,0.3,0.4,0.5)	
Ignition (CE_1_)	Expert 1	Expert 2	Expert 3
	(0.1,0.3,0.4)	(0,0.2,0.3,0.4)	(0.1,0.2,0.4)
	Expert 4	Expert 5	
	(0.1,0.2,0.3,0.5)	(0.1,0.2,0.3)	
Ventilation(CE_2_)	Expert 1	Expert 2	Expert 3
	(0.1,0.3,0.4,0.5)	(0.1,0.2,0.3,0.4)	(0,0.1,0.2,0.3)
	Expert 4	Expert 5	
	(0,0.1,0.3,0.4)	(0.1,0.2,0.3,0.4)	
Alarm(CE_3_)	Expert 1	Expert 2	Expert 3
	(0.1,0.2,0.3,0.5)	(0.1,0.2,0.3,0.4)	(0,0.1,0.3,0.4)
	Expert 4	Expert 5	
	(0,0.1,0.2)	(0,0.1,0.2,0.3)	

**Table 9:** FPS, FFR and Occurrence Probability Calculations of Operational Error and Multiple Failure.

Event	Aggregated fuzzy numbers	FPS	FFR (d^-1^)	Occurrence Probability of failure (in 1 year)
Flange is not tightly clipped (B_15_)	(0.1339, 0.2339, 0.3339, 0.4769)	0.2966	8.542e-4	2.679e-1
Operational error (B_19_)	(0.1661, 0.2846, 0.3416, 0.4831)	0.3202	1.103e-3	3.314e-1
V-4 is not closed (B_21_)	(0.1154, 0.2815, 0.3430, 0.4661)	0.2990	8.773e-4	2.740e-1
Ignition (CE_1_)	(0.0831, 0.2154, 0.2554, 0.3970)	0.2383	4.080e-4	1.384e-1
Ventilation (CE_2_)	(0.0584, 0.1738, 0.2969, 0.3969)	0.2309	3.661e-4	1.251e-1
Alarm (CE_3_)	(0.0323, 0.1323, 0.2277, 0.3431)	0.1845	1.675e-4	5.931e-2

### Occurrence Probability of LE and OE Updating

Occurrence probability of LE gas leakage and OEs were determined by Eqs 1 through 3, and the occurrence probabilities of them were listed in Table 10.

**Table 10:** Occurrence Probability of Gas Leakage and OEs.

Symbol	Occurrence Probability (in 1 year)
Gas Leakage	6.702e-1
OE_1_	7.634e-2
OE_2_	4.813e-3
OE_3_	1.092e-2
OE_4_	6.883e-4
OE_5_	4.753e-1
OE_6_	2.997e-2
OE_7_	6.796e-2
OE_8_	4.285e-3

## Discussion

The occurrence probability of gas leakage in one year was 6.702e-1 (Table 10), and occurrence probabilities of accidents would be reduced when ventilation and alarm system were present and functional. However, present and functional ventilation or alarm system can't avoid minor accidents, their occurrence probabilities like OE_3_ and OE_6_ still remained relatively high. Although ventilation and alarm system were necessary to lessen the impact of gas leakage in biomass gasification system. But the key to avoid accidents was reducing the occurrence probability of gas leakage. Thus, the critical nodes of causes for gas leakage was determined by BN-bow-tie analysis, and the corresponding safety measures were proposed according to the critical nodes.

### Confirming the Critical Nodes of Causes

To find the critical nodes of causes, the importance measures of each event was calculated by Eqs 4 through 7 (Table 11). The rank of events based on importance measures was obtained by the methods listed below.

1. If the amount of events was “*n*”, the normalized weighting R* was calculated by the importance measures *I*_*i*_ and Eq 21.
  $$R^{*}=\frac{I_{i}}{\sum_{i=1}^{n}I_{i}}\times 100$$ (21)
2. After the normalized weighting R* of each importance measure was calculated, the total weighting $R_{\#}^{*}$ was calculated by Eq 22.
  $$R_{\#}^{*}=R_{BM}^{*}+R_{RAW}^{*}+R_{FV}^{*}$$ (22)
3. Finally, events were ranked from maximum to minimum by total weighting $R_{\#}^{*}$, and the critical nodes of causes was found by the rank. The results are shown in Table 12.

**Table 11:** Calculations of Importance Measures.

Symbol	Importance Measure		
	BM	RAW	FV
B_1_	3.878e-1	8.662e-2	8.662e-2
B_2_	3.310e-1	1.832e-3	1.832e-3
B_3_	3.400e-1	1.529e-2	1.529e-2
B_4_	4.224e-1	1.383e-1	1.383e-1
B_5_	3.303e-1	7.736e-4	7.743e-4
B_6_	3.305e-1	1.183e-3	1.183e-3
B_7_	3.304e-1	9.055e-4	9.056e-4
B_8_	3.300e-1	3.413e-4	3.417e-4
B_9_	3.305e-1	1.122e-3	1.123e-3
B_10_	3.301e-1	5.285e-4	5.297e-4
B_11_	3.299e-1	1.735e-4	1.746e-4
B_12_	3.312e-1	2.149e-3	2.150e-3
B_13_	3.346e-1	7.158e-3	7.159e-3
B_14_	3.311e-1	2.055e-3	2.056e-3
B_15_	4.504e-1	1.800e-1	1.800e-1
B_16_	0	0	0
B_17_	1.000e-5	7.961e-11	0
B_18_	0	0	0
B_19_	0	0	0
B_20_	0	0	0
B_21_	4.542e-1	1.857e-1	1.857e-1

**Table 12:** Rank of Events.

Rank	Symbol	Normalization Weighting (R*)			Total Weighting ($R_{\#}^{*}$)
		$R_{BM}^{*}$	$R_{RAW}^{*}$	$R_{FV}^{*}$	
1	B_21_	7.976	29.751	29.750	67.477
2	B_15_	7.910	28.846	28.845	65.601
3	B_4_	7.418	22.156	22.156	51.730
4	B_1_	6.810	13.878	13.878	34.566
5	B_3_	5.971	2.450	2.450	10.871
6	B_13_	5.875	1.147	1.147	8.169
7	B_12_	5.816	0.344	0.344	6.504
8	B_14_	5.815	0.329	0.329	6.473
9	B_2_	5.812	0.293	0.294	6.399
10	B_6_	5.805	0.189	0.190	6.184
11	B_9_	5.804	0.180	0.180	6.164
12	B_7_	5.802	0.145	0.145	6.092
13	B_5_	5.800	0.124	0.124	6.048
14	B_10_	5.797	0.085	0.085	5.967
15	B_8_	5.795	0.055	0.055	5.905
16	B_11_	5.793	0.028	0.028	5.849
17	B_17_	≈0	≈0	0	≈0
18	B_16_	0	0	0	0
19	B_18_	0	0	0	0
20	B_19_	0	0	0	0
21	B_20_	0	0	0	0
Total		100	100	100	100

The total weighting of B_21_ (V-4 is not closed), B_15_ (flange is not tightly clipped), B_4_ (wear of VP) and B_1_ (VP seal failure) was much higher than others; they were the critical nodes of causes. Because accidents were mainly caused by these nodes, the occurrence probabilities of accidents could be reduced effectively by implementing corresponding safety measures. If B_21_, B_15_, B_4_ and B_1_ were implemented with measures to ensure safety, the occurrence probabilities was reduced to 1/10.3 of the original values (Table 13).

**Table 13:** Occurrence Probabilities of Accidents when Critical Nodes are Ensured Safety.

Symbol	Occurrence Probability (in 1 year)
Gas Leakage	6.527e-2
OE_1_	7.435e-3
OE_2_	4.688e-4
OE_3_	1.063e-3
OE_4_	6.703e-5
OE_5_	4.628e-2
OE_6_	2.918e-3
OE_7_	6.618e-3
OE_8_	4.173e-4

### Providing Safety Measures for Critical Nodes

B_21_ (V-4 is not closed) and B_15_ (flange is not tightly clipped) were operational errors. To eliminate these errors, a safety check was added to make sure that flange was tightly clipped before production. Additionally, the V-4 manual valve was replaced with a self-closing valve. Both B_4_ (wear of VP) and B_1_ (VP seal failure) are facilities failures; the VP safety checks should be improved and the VP seals replaced at regular intervals.

## Conclusions

1. In biomass gasification system, facilities failure data can be obtained from standard reliability data sources, and operational error data can be confirmed by fuzzy methods based on expert judgment. These reliability data can be used to make probabilistic safety assessment (PSA) of biomass gasification system.
2. Bow-tie analysis was employed to evaluate gas leakage from biomass gasification stations. When ventilation and alarm systems were present and functional, the occurrence probabilities of accidents caused by gas leakage were reduced, but they were inefficient in reducing the occurrence probabilities of minor accidents. Therefore, the occurrence probability of gas leakage must be lessened to reduce the exposure to associated accidents caused by gas leakage.
3. By mapping bow-tie analysis into BN (BN-bow-tie), the critical nodes of accidents causes were identified. These critical nodes of gas leakage were as follows: V-4 is not closed, the flange is not tightly clipped, wear of VP, and VP seal failure. If safety measures were implemented at these nodes, the occurrence probabilities of accidents were reduced to 1/10.3 of the original values.
4. To reach the safety goal, safety checks should be added. The manual V-4 valve should be replaced with a self-closing valve, and the VP seals should be replaced periodically.
